# Non-verbal communication of compassion: measuring psychophysiologic effects

**DOI:** 10.1186/1472-6882-11-132

**Published:** 2011-12-20

**Authors:** Kathi J Kemper, Hossam A Shaltout

**Affiliations:** 1 Social Science and Health Policy, Pediatrics, Center for Integrative Medicine, and Hypertension and Vascular Research Center; Wake Forest University School of Medicine; Winston-Salem, NC, USA; 2 Hypertension and Vascular Research Center, Center for Integrative Medicine, Department of Obstetrics and Gynecology; Wake Forest University School of Medicine; Winston-Salem, NC, USA

## Abstract

**Background:**

Calm, compassionate clinicians comfort others. To evaluate the direct psychophysiologic benefits of non-verbal communication of compassion (NVCC), it is important to minimize the effect of subjects' expectation. This preliminary study was designed to a) test the feasibility of two strategies for maintaining subject blinding to non-verbal communication of compassion (NVCC), and b) determine whether blinded subjects would experience psychophysiologic effects from NVCC.

**Methods:**

Subjects were healthy volunteers who were told the study was evaluating the effect of time and touch on the autonomic nervous system. The practitioner had more than 10 years' experience with loving-kindness meditation (LKM), a form of NVCC. Subjects completed 10-point visual analog scales (VAS) for stress, relaxation, and peacefulness before and after LKM. To assess physiologic effects, practitioners and subjects wore cardiorespiratory monitors to assess respiratory rate (RR), heart rate (HR) and heart rate variability (HRV) throughout the 4 10-minute study periods: Baseline (both practitioner and subjects read neutral material); non-tactile-LKM (subjects read while the practitioner practiced LKM while pretending to read); tactile-LKM (subjects rested while the practitioner practiced LKM while lightly touching the subject on arms, shoulders, hands, feet, and legs); Post-Intervention Rest (subjects rested; the practitioner read). To assess blinding, subjects were asked after the interventions what the practitioner was doing during each period (reading, touch, or something else).

**Results:**

Subjects' mean age was 43.6 years; all were women. Blinding was maintained and the practitioner was able to maintain meditation for both tactile and non-tactile LKM interventions as reflected in significantly reduced RR. Despite blinding, subjects' VAS scores improved from baseline to post-intervention for stress (5.5 vs. 2.2), relaxation (3.8 vs. 8.8) and peacefulness (3.8 vs. 9.0, P < 0.05 for all comparisons). Subjects also had significant reductions in RR (P < 0.0001) and improved HRV (P < 0.05) with both tactile and non-tactile LKM.

**Conclusion:**

It is possible to test the effects of LKM with tactile and non-tactile blinding strategies; even with blinding in this small preliminary study, subjects reported significant improvements in well-being which were reflected in objective physiologic measures of autonomic activity. Extending compassion is not only good care; it may also be good medicine.

**Trial registration number:**

US National ClinicalTrials.gov registration number, NCT01428674

## Background

Compassionate communication is the cornerstone of clinical care. Although most research has focused on outcomes of verbal communication, there is little research on non-verbal communication, which experts estimate is the predominant form of communication [1-4]. Positive non-verbal communication (NVC) can have powerful clinical benefits, but the precise mechanisms by which NVC of compassion (NVCC) benefits patients have not been fully explored [5]. Better understanding of the psychophysiologic mechanisms by which clinicians' NVCC (both non-verbal/non-tactile, and non-verbal/tactile communication) benefits patients could lead to innovations in educational strategies to maximize these benefits for a variety of clinicians, including psychotherapists, physicians, massage therapists, nurses, acupuncturists, and others who interact directly with patients. It may also lay evidence-based groundwork to develop brief training interventions for parents or other caregivers to learn to effectively extend compassion and reduce stress in hospitalized or palliative care patients.

Studies in this area are challenging because subjects' expectations can strongly influence their response to an intervention [6-10]. That is, the expectation that one will receive benefit from a clinician may influence the impact of the clinician's actions or words [11-13]. When evaluating the effects of non-verbal communication of compassion (NVCC), minimizing subjects' expectations is critical for understanding its true effects. Although it is optimal for subjects to be blind to the NVCC, published protocols establishing effective blinding strategies are scarce; it would be useful to test two distinct strategies that might be employed under different experimental and clinical conditions. A non-tactile strategy would be relevant for studies of psychotherapists or social workers, while a tactile strategy would be relevant for studies of massage therapists and other body workers, acupuncturists, and other therapies or diagnostic maneuvers involving tactile contact.

We wished to test a form of NVCC associated with a calm face and lower respiratory rate. One type of mental activity that generally produces these effects in the practitioner is meditation [14-25]. Among the many types of meditation, *loving-kindness-focused meditation (LKM) *is attractive because the desire for increased well-being and decreased suffering is an explicit part of health care, regardless of the modality. In LKM, the practitioner focuses on the intention that another person experiences safety, health, peace, happiness, and freedom from suffering [26-28]. Although LKM has distinct physiologic effects on the practitioner [26,28-32], we are unaware studies evaluating the effect of one person practicing LKM on another person in the same room who is not meditating and blind to the practitioner's activity.

Therefore, to lay the groundwork for definitive clinical trials, we performed a preliminary study to answer questions about the feasibility of evaluating the psychophysiologic impact of NVCC on subjects blind to the intervention. Specifically, we wished to know:

Were two different strategies for blinding subjects to LKM intervention feasible? That is, would subjects remain blind and would the LKM provider be able to maintain LKM under these experimental conditions (non-tactile and tactile strategies)?

Would blind exposure to LKM be associated with any measurable changes subjects' psycho-emotional state and/or autonomic nervous system (ANS) activity?

The primary purpose of this study was to determine the feasibility of two different strategies for NVCC of compassion using LKM meditation in subjects blind to the study interventions. Because it was a preliminary feasibility study, we did not expect to find statistically significant differences, but we wished to observe changes in relaxation, peacefulness, stress, respiratory rate (RR), and vagal tone, reflected in increased heart rate variability (HRV), particularly High Frequency (HF) power in the power spectral analysis of HRV in order to calculate sample sizes for definitive studies.

## Methods

### Subjects

To address these questions, we recruited a convenience sample of adult volunteers at Wake Forest Baptist Health's Center for Integrative Medicine over 2 months prior to a grant deadline to obtain feasibility data for the proposal. Subjects were eligible if they were healthy and not taking medication for asthma, hypertension, or diabetes, or prophylactic medications, such as beta blockers for migraine headaches. Subjects were told the project was evaluating the effect of time, touch, and attention on autonomic nervous system (ANS) function, and were asked to remain quiet throughout the study period to avoid affecting ANS activity. Electronic devices such as cellular telephones and pagers were turned off during the study period to avoid disruptions. For this study, subjects served as their own control with baseline and follow-up periods compared with two NVCC periods.

### Practitioner

Because previous studies have found differences between novice and experienced practitioners [32,33], we selected a practitioner with over 40 years of meditation experience. This senior staff member had engaged in LKM meditation daily for more than 5 years. Like the subjects, she was also a healthy female who was not taking beta blocking medications or any other medications affecting respiratory or heart rate.

### Procedure

After informed consent, subjects completed brief questionnaires on demographics and 10 point self-report visual analog scales (VAS) for stress, relaxation, and peacefulness. After the interventions and the post-intervention rest period, subjects completed the same VAS.

After completing the baseline VAS, the subject and practitioner put on BioPac^® ^Bioharness belts to collect continuous autonomic data for the 40 minute study. The Bioharness is an FDA-approved continuous ambulatory monitoring system which collects data on pulmonary, cardiac, and skin temperature measures with a sample rate set at 250 Hz. It measures rib cage excursions for respiratory rate (RR) and an ECG to measure interbeat intervals to calculate heart rate variability (HRV); a built in thermometer measures skin temperature on the chest. The lightweight device is worn under normal clothing around the chest. It is machine washable, comfortable, and includes patented software which decrypts and processes the recorded data, and allows transformations of the data for export into statistical software.

**Figure 1:**
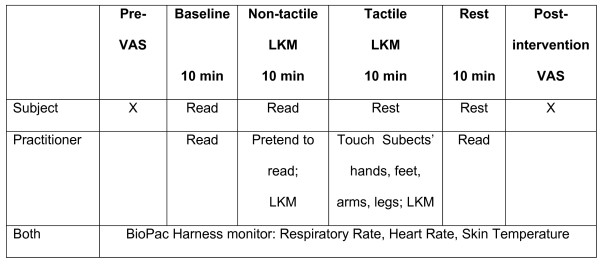
**Study Visit**. LKM- Lovingkindness meditation VAS- visual analog scales for stress, relaxation, peacefulness NVCC - Non-verbal communication of compassion.

Each subject underwent a standard study sequence consisting of four consecutive periods of 10 minutes each at one study visit: Baseline; Non-tactile LKM; Tactile-LKM; and Rest. (see Figure 1) If the purpose of the study had been to compare the effects of the two strategies, a random order separated by a rest period would be preferable, but for the purposes of this preliminary project (establishing feasibility), a standard sequence was used to minimize confusion among participants and staff.

During the Baseline control period, subject and practitioner sat in the same room approximately 8 feet apart and read quietly from an emotionally neutral textbook (*MindSight *by Daniel Siegel). During non-tactile NVCC, subjects continued to read; the practitioner practiced LKM while pretending to read (eyes open and turning pages as the subject turned pages). The practitioner focused on one phrase each breath for four breaths: 1) "may you be safe and secure", 2) "may you be healthy, comfortable and filled with vitality", 3) "may you be peaceful and happy", 4) "may you be free from suffering." The practitioner repeated the series of four breaths/lovingkindness thoughts throughout the 10 minute intervention. During the tactile NVCC strategy, subjects were told we were testing the effect of light touch on the ANS; we asked the subject to sit quietly while the practitioner lightly touched her feet, legs, hands, arms, and shoulders. During tactile LKM, the practitioner again focused on the four lovingkindness phrases/breaths with her eyes open, using the 4 phrase meditation as a timer so that each body part was touched for the time it took to complete 4 breaths before moving to a new area. Subjects sat in a straight back chair with arm rests, while the practitioner sat on a low rolling stool to facilitate moving to touch each side of the body. The practitioner was asked to keep her eyes open during both LKM interventions and avoid making eye contact with the subject. During the 4^th ^period, subjects rested quietly without reading and the practitioner remained in the room, quietly reading.

### Outcomes

To assess blinding, at the end of the forty minute study period, the experimenter asked each subject open ended questions about what occurred during each study period. For example, what were you doing during the first reading period? What was the practitioner doing during the first reading period? What were you doing during the second reading period? What was the practitioner doing during the second reading period? What were you doing when the practitioner was touching you? What was the practitioner doing when she was touching you? What were you doing during the final rest period? What was the practitioner doing during the final rest period?

Respiratory rate (RR) was selected as the primary outcome for ANS function because it is easily observed and readily measured; it is sensitive to affective states and meditation [34,25-40]; and responds quickly to stimuli [41-44]. For analysis of RR, we downloaded the BioHarness data to an MS Excel database, analyzing median samples for 15 second epochs. Similar analyses were conducted for secondary outcomes of heart rate (HR) and peripheral skin temperature (Temp).

For heart rate variability (HRV) analyses, two parameters of interest were a) Total Power which reflects overall HRV, and activity in the high frequency (HF) power band of the power spectrum analysis, which reflects vagal tone in short-term recordings. BioPac^® ^-acquired files were re-sampled at rate of 1 HZ, and the interbeat intervals (IBI) between successive RR-peaks files were analyzed using Nevrokard^® ^- HRV software (Medistar, Ljubljana, Slovenia) using standard algorithms. Outliers were identified as all interbeat intervals (IBIs) demonstrating a 30% difference from the mean of the previous four samples, and were removed from the data set. Power spectral densities for IBI were computed by Fast Fourier Transform (FFT), integrated over the standard frequency range (VLF; < 0.04 Hz), (LF; 0.04-0.15 Hz) and (HF; 0.15-0.4 Hz), and a Hanning window was applied using standard methods [45-48].

As a secondary question, we explored practitioner-subject correlation in RR.

### Analysis

Analyses focused on descriptive statistics and impact on RR and VAS scales using SAS 9.2 (SAS Institute, Cary). Quantitative variables were summarized by means and standard deviations. The secondary outcome measures of subjects' HR, HRV, and skin temperature (Temp) were analyzed using these same methods. In addition to these models where each outcome is taken as a single value, we used a mixed models approach to allow for the repeated assessments of outcomes in each subject that occur during the study. In this model, participants are treated as random effects and overall dose and delivery estimates were calculated accounting for the repeated measurements. This approach allowed us to detect smaller differences than the ANCOVA models described above since they provide a more precise estimate of the outcome effects. All tests were two-sided with a type 1 error rate of 5%.

This study was approved by the Institutional Review Board of Wake Forest School of Medicine.

## Results

Because the study was designed primarily as a feasibility trial, we aimed for complete data collection on a convenience sample of 5 subjects; enrollment was closed when six subjects were recruited to provide an extra subject as back-up for missing data. All subjects were women; the mean age was 43.6 ± 10.8 years. None had any known cardiopulmonary conditions and none were taking beta-blocking medications.

### Feasibility

Blinding was maintained, i.e., all subjects reported that the practitioner was reading during baseline, reading during the non-tactile LKM and touching during the tactile LKM. One subject reported that she thought the practitioner "might be meditating" (during non-tactile LKM) because "the room got really quiet", but when she looked, the practitioner was turning a page of the book, so "she must be really reading". The practitioner maintained a meditative state during both tactile and non-tactile LKM interventions as reflected by her verbal report and in changes in RR. On average, the practitioner's RR fell from 15.9 breaths per minute (bpm) at baseline to 6.5 bpm during non-tactile LKM and 8.2 bpm during tactile LKM, returning to 13.7 bpm during the post-intervention rest (P < 0.01 for both LKMs vs. baseline and rest).

### Observed Psychophysiologic Effects

As shown in Table 1, subjects reported decreases in stress and increases in relaxation and peacefulness after exposure to NVCC. For example, subjects' average relaxation increased from 3.8 at baseline to 8.8 on a 0 to 10 VAS after the NVCC interventions (P < 0.05.) Subjects also had significant autonomic changes in the expected direction with NVCC. Subjects' average RR fell from baseline more than 4 bpm during non-tactile-LKM (P < 0.001) and more than 3 bpm during tactile-LKM (P < 0.001), and remained an average of 1.4 bpm below baseline values for the post-intervention rest period (P < 0.01). Heart rate fell for both tactile and non-tactile LKM interventions (P < 0.05). Overall HRV, reflected in Total Power, increased significantly for both interventions compared to baseline (P < 0.05). High Frequency (HF) power, reflecting primarily parasympathetic or vagal activity, increased from baseline for both tactile and non-tactile LKM, and declined to near baseline levels during the post-intervention rest periods (P < 0.05 for both). Peripheral skin temperature increased significantly over time (P < 0.001); however, its steady increase throughout the study leads to concern that the device may simply take 40 minutes or more to reach an equilibration with skin temperature after being cleaned and then stored at room temperature.

**Table 1:** Changes from Baseline in Average Autonomic Nervous System Activity and Psycho-emotional State for Non-tactile NVCC, Tactile NVCC, and Follow-up Rest in 6 healthy subjects

Measure	1. Baseline Mean (± SEM) 10 minutes	2. Change with Non-tactile NVCC Mean (± SEM) 10 minutes	3. Change with Tactile NVCC Mean (± SEM) 10 minutes	4. Rest Mean (± SEM) 10 minutes
***Autonomic Data ***				

**RR** **(breaths per minute)**	15.33 (± 1.67)	- 4.34 (± 0.18)***	-3.55 (± 0.11) ***	-1.36 (± 0.15) ***

***Other Autonomic Measures***				

**HR** **(beats per minute)**	73.93 (± 0.22)	-1.63 (± 0.16)***	-2.94 (± 0.32)***	-4.40 (± 0.34)***

**HRV: Total Power (TP)**	761.8 (± 19.05)	+225.7 (± 39.7)*	+436.1 (± 64.6)**	+ 206.6 (± 49.6)

**HRV: High Frequency**	41.02 (± 1.14)	+18.4 (± 2.7)*	+12.9 (± 3.6)*	+5.2 (± 2.7)

**Skin Temp (Centigrade)**	33.6 (± 0.004)	+1.18 (± 0.01)***	+ 1.95 (± 0.046)***	+2.28 (± 0.02)***

***Psycho-emotional state: Visual analog scales with numeric anchors:*** *0 = not; 10 = very (relaxed, stressed, peaceful)* *(*± *Standard Deviation)*				

**Relaxed**	3.8 (± 2.4)	Post NVCC: 8.8 (± 1.2) *		

**Stressed**	5.5 (± 3.2)	Post NVCC: 2.2 (± 1.7)*		

**Peaceful**	3.8 (± 0.8)	Post NVCC: 9.0 (± 0.9)**		

Non-tactile NVCC - the practitioner practiced loving-kindness meditation while pretending to read a book

Tactile NVCC - the practitioner practiced loving-kindness meditation while touching the subject on the hands, arms, shoulders, knees, and hips "to test the effects on the autonomic nervous system"

* P < 0.05; ** P < 0.01; *** P < 0.0001

**Figure 2:**
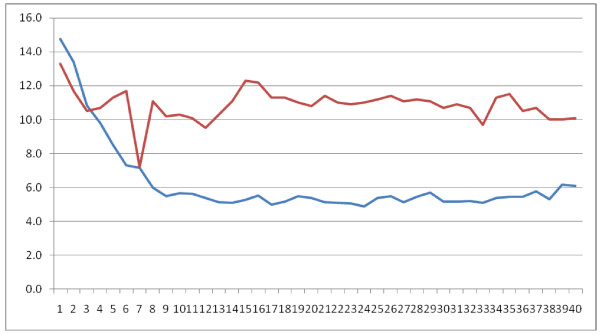
**Average RR for Practitioner (blue) and Subjects (red) during Non-tactile NVCC over 10 minute intervention (40 15 second averages)**. Correlation, r = 0.55, P < 0.001.

In the analysis exploring the correlation between practitioner and subjects' respiratory rates, there was a significant correlation during non-tactile LKM (r = 0.55, P < 0.001). As the practitioner began to meditate, her RR fell rapidly, reaching the nadir of < 6 bpm at 2.5 minutes, which was generally maintained until the end of non-tactile LKM. Subjects' RR also fell over the first 2.5 minutes, after which it stayed between 10 and 12 bpm for the remainder of the period and remained lower than baseline during tactile LKM. (Figure 2) There were no significant correlations between practitioner and subject RR during rest, tactile LKM, or post-intervention rest periods.

## Discussion

Although compassion is a cornerstone of clinical care, there is a gap in research exploring the feasibility of measuring its direct psychophysiologic impact on subjects blind to the practitioner's intention. This preliminary study demonstrates that it is feasible to conduct blinded studies of the interpersonal effects of one form of NVCC, lovingkindness meditation (LKM) and provides preliminary evidence of significant psychophysiologic effects: increased relaxation, peacefulness, and heart rate variability, and decreased stress and respiratory rate.

The practitioner's decrease in RR during LKM practice in this study is consistent with earlier studies showing significant decreases in RR with meditation [34,49,50]. Furthermore, these data suggest that in addition to having beneficial psychological and physiologic effects for practitioners [19,27,31,51-53], practicing compassion can also have physiologic and psychological effects on others, even when they are blind to the intervention.

Modern cultures place a premium on verbal communication. Thus, it is challenging to design experimental intervention strategies in which subject and practitioner are silently together over 40 minutes without arousing discomfort. We tested two strategies for blinding subjects to the intervention (reading and light touch) because each has pros and cons and may be better suited for clinical studies in different settings (e.g., psychotherapy vs. massage therapy). Practitioner "reading" is a useful blinding strategy because it is a common activity and generally results in a comfortable silence when two people are reading together; however, it is possible that subjects may be so absorbed in reading that they pay little attention to the practitioner's non-verbal cues, limiting their effects. It may also be challenging for less experienced practitioners to keep eyes open and focused on a page, turning pages from time to time while engaged in LKM, but it is feasible with practice. The light touch strategy allows the subject closer, undistracted observation of the practitioner, but may be uncomfortable outside of an established clinical context or relationship. The suggestion that the purpose of touch is to elicit autonomic activity is different from the suggestion that the purpose of touch is to comfort or heal, and suggestions themselves may have potent psychophysiologic effects [6,54]. These data suggest that both non-tactile and tactile strategies are feasible for blinding subjects to LKM; future clinical research can choose the strategy best suited for their study, e.g., non-tactile "reading" for a study of the effects of NVCC during psychotherapy or educational interventions vs. tactile "touch eliciting effects on autonomic function" for studies of the effects of NVCC during massage or bodywork.

What conceptual model or theory might explain the expected benefits of NVCC? The theory of *mirror neurons *guided our study. Mirror neurons in the central nervous system (CNS) fire when an animal (or human) acts *and *when it simply *observes *another take that same action. These neurons unconsciously mirror other's behavior. Mirror neurons also fire in response to observed emotions [55-58]. Thus, individuals who are calm, e.g., who have a calm facial expression and slower, deeper respiratory rate (RR), may, via mirror neurons, trigger a sense of calm and perhaps a slower RR in another person [57,59-63]. This study focused on mirrored RR because it is objective and easily measured. Our data are consistent with mirror neuron system (MNS) theory and studies showing that non-verbal communication of emotion and intention occur through the MNS outside of conscious awareness because our subjects showed psychophysiologic changes in the expected direction even though they were blind to the actual intervention [57,61,64,65].

### Limitations

Because this study was focused primarily on feasibility, it had a small convenience sample of healthy women in a fixed sequence design. Given the small sample size and low statistical power, we were surprised to find statistically significant effects on both self-reported psychological outcomes and objective physiologic measures. Although these results are intriguing, future studies should include a larger number of more diverse subjects in a randomized intervention order. Because empathy has been linked to MNS responses [57,59,61,64], future studies should also test whether subjects with varying degrees of empathy have varying autonomic and psycho-emotional effects with NVCC.

This study explored only RR signals although NVCC may include changes in facial expression, operating through different MNS pathways. Future studies may focus on facial expressions as cues for NVCC [66-69]. For such studies, video recordings of the practitioner and subject would be desirable. Furthermore, only one type of meditation was tested in this study. Some research suggests that different types of meditation have different physiologic impact on practitioners which may be translated into different nonverbal cues [14,19,26,53,70,71]. Future studies may compare the effects of lovingkindness meditation with other types of meditation practice.

The "dose" of optimal exposure to NVCC is unknown. This study used two 10 minute 'doses' of exposure to LKM, and although we noted an onset of effect on RR within that time, future studies should compare the impact of different durations LKM intervention.

The timing and duration of effects are unknown. We asked subjects to rate stress, relaxation, and peacefulness after both LKM interventions (20 minutes) and the post-intervention rest period (10 minutes) with simple VAS scales rather than standardized psychological assessment tools. Since RR tended to return to baseline levels by the end of the post-intervention period, it may be preferable to ask subjects to complete self-report measures immediately after each intervention and to use standardized measures. If more than one type of LKM is provided, subjects should receive them in random order to minimize sequence effects, and there should be separate measurements and a "wash out" period between interventions to identify the unique effect of each intervention.

This study used an experienced LKM practitioner, and it is unclear whether the same effects would be found with practitioners who had brief training or little experience. Most studies comparing long-term with novice meditation practitioners have noted differences [32,33,72,73]. Clinically, it would be useful to know, for example could parents of anxious, hospitalized children be trained to practice LKM for and with their child, reducing the child's anxiety and stress? Could loved ones of palliative care patients be trained to extend LKM, and what impact would this have on patients' well-being, clinical outcomes, and satisfaction with care? How much LKM practice is sufficient to have a sustained and meaningful impact on others?

This study intentionally omitted any positive suggestion about the impact of NVCC on subjects. It is of clinical importance to understand how the benefits observed here with blind intervention could be augmented by positive suggestions.

For this study, the intervention used sham activities (reading and light touch), but did not require the practitioner to actually engage in another productive activity (e.g., checking a pulse, taking a history) during LKM. It would be worthwhile to determine the feasibility and impact of simultaneously engaging in LKM and another meaningful clinical activity. It would also be worthwhile to understand the effects of clinicians engaging in LKM prior to or between clinical activities on patients' sense of well-being and autonomic activity.

Temperature increases were noted throughout all four study periods, which could be consistent with relaxation or simply with the device equilibrating from room temperature to body temperature over time [74,75]. Future studies should use a control intervention or longer warm up period to ensure the device has reached a stable temperature before starting data collection.

Although the hypothesized mechanism for the effects of NVC observed in this study is the mirror neuron system, this study did not directly measure CNS activity, but focused instead on feasibility, self-report, and autonomic measures. Now that feasibility has been established and the data suggest psychophysiologic effects, it would be worthwhile to further test the mirror neuron theory of NVCC through imaging studies.

## Conclusion

It is feasible to study the interpersonal effects of non-verbal compassionate communication on the autonomic nervous system and psycho-emotional state while maintaining subject blinding to the intervention using strategies appropriate for clinical situations including either tactile (e.g., massage) or non-tactile (e.g., psychotherapy) interactions. Furthermore, despite the small sample size in this preliminary study, the intervention (lovingkindness meditation with an experienced practitioner) was associated with significant changes in subjects' ANS as well as significant increases in relaxation and peacefulness even though subjects remained blind to the nature of the intervention. Future projects evaluating the impact of clinician or parent training can build on this work to explore the underlying mechanism and the clinical consequences of the interpersonal effects of communicating compassion.

## Abbreviations

**ANS**: autonomic nervous system; **CNS**: central nervous system; **HF**: high frequency, a measure of heart rate variability from the power spectral calculation of HRV, often associated with vagal tone; higher values are associated with relaxation; **HR**: heart rate, measured in beats per minutes; **HRV**: heart rate variability; the variability in the interbeat interval; **IBI**: interbeat interval; **LKM**: lovingkindness meditation; **LF**: low frequency, a measure of heart rate variability from the power spectral calculation of HRV; **NVC**: non-verbal communication; **NVCC**: non-verbal communication of compassion; **RR**: respiratory rate, measured in breaths per minute; **SDNN**: standard deviation of the interbeat interval; often used as a proxy for HRV with higher values reflecting greater well-being; **Temp**: peripheral skin temperature; **TP**: Total Power, an overall measure of heart rate variability from the power spectral calculation of HRV; often used as a proxy for HRV with higher values reflecting greater well-being; **VLF**: Very low frequency, a measure of heart rate variability from the power spectral calculation of HRV.

## Declaration of Competing interests

The authors declare that they have no competing interests.

## Authors' contributions

KJK conceived of the project, developed the protocol, recruited subjects and practitioner, supervised the study, downloaded data, and analyzed the VAS, RR, HR, and Temperature data. Dr. Kemper wrote the drafts of the manuscript and revised them with input from Dr. Shaltout.

HAS analyzed the HRV data, double-checked the other data analyses, wrote the HRV sections of the methods and results, and participated in revising and clarifying the paper.

Both authors read and approved the final manuscript.

## Pre-publication history

The pre-publication history for this paper can be accessed here:

http://www.biomedcentral.com/1472-6882/11/132/prepub
